# Current research progress on the use of traditional Chinese medicine in the treatment of diabetic foot ulcers

**DOI:** 10.3389/fendo.2025.1637128

**Published:** 2025-08-22

**Authors:** Yinfeng Xia, Ping Wu, Yongsong Chen, Zhiyong Chen

**Affiliations:** Department of Burns, Plastic Surgery and Cosmetology, Chongqing University Fuling Hospital, Chongqing University, Chongqing, China

**Keywords:** diabetic foot ulcers, traditional Chinese medicine, wound, diabetes mellitus, wound healing

## Abstract

Diabetic foot ulcers represent a significant complication of diabetes mellitus, presenting substantial challenges due to their intricate pathogenesis, which encompasses neuropathy, vasculopathy, chronic inflammation, and biofilm-associated infections. Despite considerable advancements in Western medical interventions, including surgical debridement, skin grafting, negative pressure wound therapy, and innovative dressings, these ulcers remain a leading cause of amputation and contribute to a substantial socioeconomic burden. Traditional Chinese medicine (TCM) has emerged as a promising adjunctive therapy, offering multi-targeted mechanisms that address oxidative stress, chronic inflammation, angiogenesis, and microbial resistance associated with diabetic foot ulcers. This review aims to examine contemporary studies on the application of TCM in the treatment of diabetic foot ulcers, evaluating its efficacy and elucidating its mechanisms of action, thereby providing a reference for clinical treatment decisions and guiding future research directions.

## Introduction

Diabetes mellitus (DM) is a chronic metabolic disorder resulting from the interplay of genetic and environmental factors, with its prevalence rising annually (1). Diabetic foot ulcers (DFUs) represent a significant complication of diabetes mellitus, typically arising from a series of metabolic dysfunctions induced by a hyperglycemic environment. This environment leads to neuropathy, vasculopathy, and ultimately neurological disease and peripheral vascular injury in the lower extremities, as well as deep tissue damage (2, 3). The incidence of diabetic foot ulcers is increasing each year, with approximately 20% of affected patients eventually requiring amputation (4–7). The complex pathogenesis and prolonged duration of diabetic foot ulcers have rendered them a substantial socioeconomic burden (8).

Presently, the primary strategies in Western medical treatment include glycemic control, infection management, removal of necrotic tissue, decompression, hyperbaric oxygen therapy, and restoration of blood flow (9–12).Interventions such as surgical debridement, skin grafting, negative pressure therapy, novel biological dressings, cytokine therapy, and stem cell transplantation have demonstrated promising outcomes (13–15). With the advancement of Traditional Chinese Medicine (TCM), it has been demonstrated to possess satisfactory efficacy in the clinical management of diabetes mellitus and its associated complications (16). Research indicates that herbal medicine serves as a safe and effective adjunctive therapy to conventional treatments for diabetic foot, facilitating the healing of diabetic foot ulcers (17, 18). However, there are currently few review articles on the treatment of diabetic foot ulcers with TCM, making it difficult to quickly obtain the latest research findings in this field. Consequently, this study aims to synthesize the current research landscape regarding the use of TCM in the treatment of diabetic foot ulcers, thereby providing a reference for future research directions.

## Pathophysiology of diabetic foot ulcers

**Figure 1:**
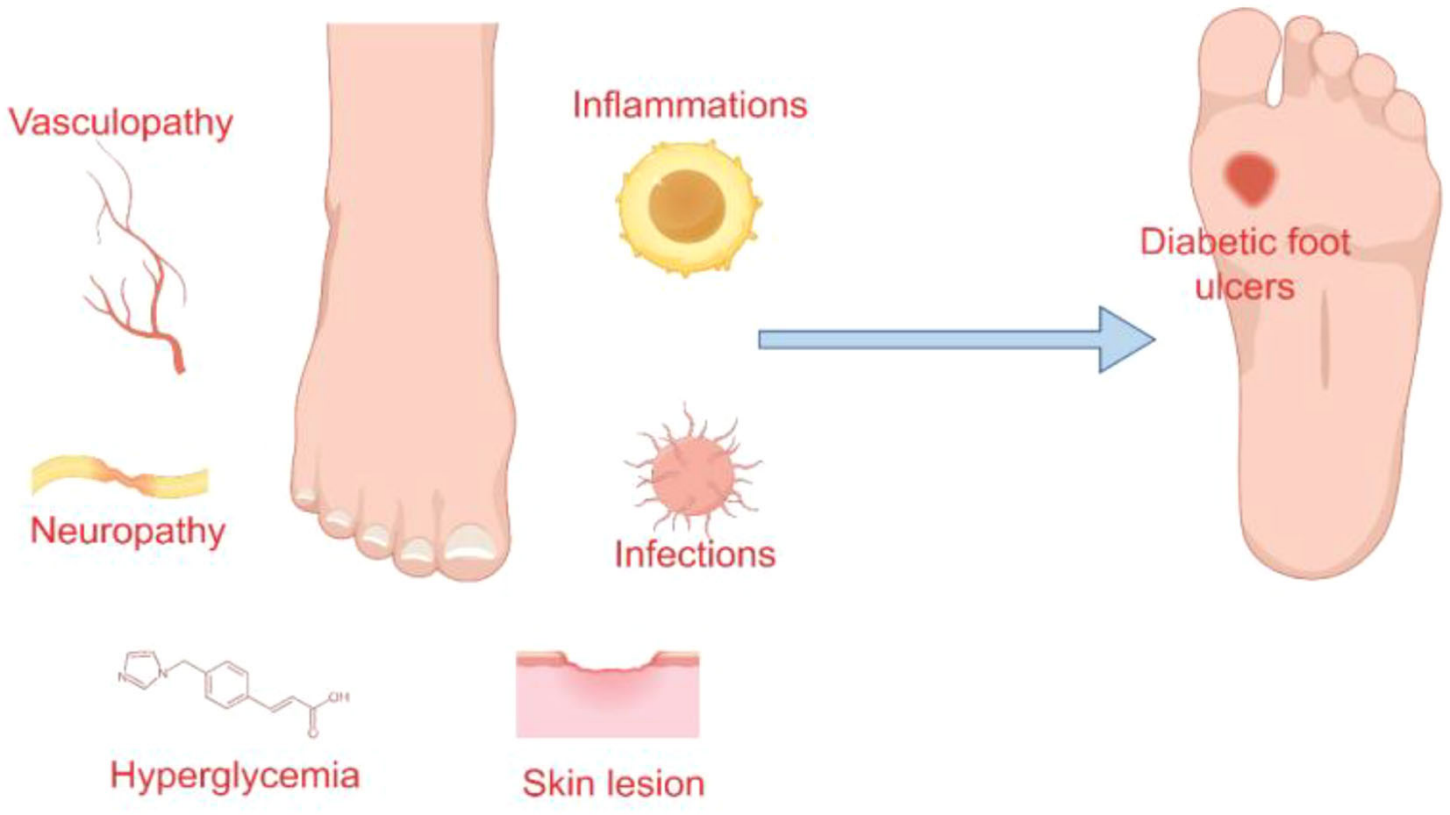
Causes of diabetic foot ulcers.

The pathogenesis of diabetic foot ulcers is predominantly linked to neuropathy, vasculopathy, and infection and inflammation (19, 20) (**Figure 1**). Neuropathy contributes to the formation of high-pressure areas on the plantar surface of the foot, particularly at the hammertoes and metatarsal heads, while diminished protective sensory function renders the skin more susceptible and less perceptible, ultimately leading to ulcer development (13).

Angiogenesis is a critical component of wound healing, with the functionality of vascular endothelial cells playing a pivotal role in this process (21, 22). The hyperglycemic and insulin-resistant microenvironment downregulates the expression of endothelial nitric oxide synthase (eNOS) and impairs the transport capacity of L-arginine across the endothelial cell membrane, consequently reducing nitric oxide (NO) production. Furthermore, elevated levels of advanced glycation end products (AGEs) can inactivate NO, while a decrease in tissue plasminogen activator (tPA) contributes to endothelial cell dysfunction (23–25). Such endothelial cell damage compromises vascular dilation, impairs blood flow and perfusion at the wound site, and ultimately delays wound healing (26). Moreover, angiogenesis is reliant on various proangiogenic factors, including vascular endothelial growth factor (VEGF-A), fibroblast growth factor (FGF-2), and platelet-derived growth factor (PDGF) (27–29). Disorders in glucose metabolism can affect the function of hypoxia-inducible factor 1-α (HIF-1α), thereby inhibiting the activity of these growth factors and adversely affecting angiogenesis (30). This results in the thickening of the basement membrane, impaired vasodilation, and compromised microcirculation, thereby exacerbating ischemia, hypoxia, and peripheral nerve damage associated with diabetic foot ulcers (31–33).

Another pathological feature of diabetic foot ulcers, particularly those that are refractory to healing, is the prolonged chronic inflammatory phase. Hyperglycemia disrupts immune function, elevates levels of inflammatory cytokines, and facilitates biofilm formation (34–36). In diabetic patients, neutrophil dysfunction impairs macrophage polarization and the release of neutrophil extracellular traps (NETs), further intensifying the inflammatory response (37, 38). The presence of macrophages with various phenotypes is crucial for wound healing, with M2 macrophages playing a pivotal anti-inflammatory role, potentially through the promotion of vascular endothelial growth factor secretion (39, 40). The prolonged polarization of M1 macrophages extends the inflammatory response (41). Furthermore, T cells and natural killer (NK) cells intensify this inflammatory process. In individuals with diabetes, there is an increased presence of pro-inflammatory T cells in the bloodstream. Within ulcers, there is an accumulation of effector T cells, while naive T cells are markedly diminished, contributing to delayed wound healing (42, 43). NK cells enhance the inflammatory response by secreting interferon-γ, perforins, and granzymes; the interferon they release also facilitates M1 macrophage polarization and neutrophil infiltration (44, 45). The continuous infiltration of macrophages and neutrophils consequently results in impaired wound healing (46). Moreover, the hyperglycemic microenvironment present in wounds creates optimal conditions for bacterial proliferation and colonization, facilitating the formation of bacterial biofilms. These biofilms contribute to increased bacterial resistance and the likelihood of infection (47, 48). The development of bacterial biofilms triggers the release of elevated levels of inflammatory cytokines by the host, thereby extending the inflammatory phase of wound healing. Chronic inflammation is recognized as an important factor in the persistence of non-healing wounds (49–51).

## Mechanisms of TCM in the treatment of diabetic foot ulcers

**Figure 2:**
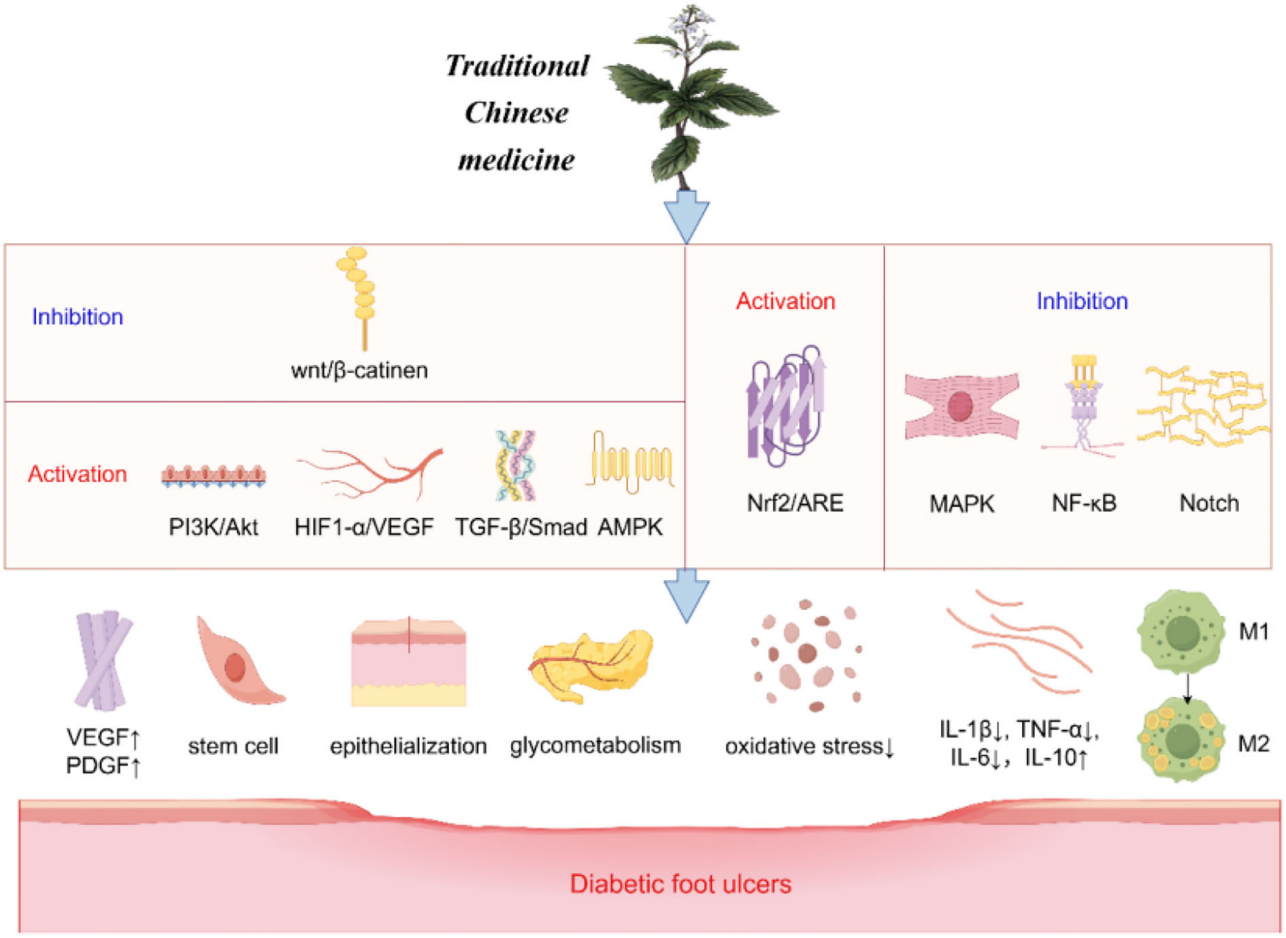
Mechanism of traditional Chinese medicine treatment for diabetic foot ulcers.

The healing process of diabetic foot ulcers encompasses several factors, including the amelioration of neurological and vascular lesions, control of oxidative stress, as well as the management of infection and inflammation (**Figure 2**).

### Promote angiogenesis

The Wingless/Integrated (Wnt) signaling pathway promotes wound angiogenesis and epithelial remodeling, among other functions, and is associated with wound healing. Closely related to this is the classic Wnt/β-catenin pathway (52). Terpenoids derived from TCM have been shown to reduce apoptosis and promote the proliferation and differentiation of epidermal stem cells via the Wnt/β-catenin signaling pathway (53, 54). The Phosphoinositide 3-kinase/Protein Kinase B (PI3K/Akt) pathway is a well-established anti-apoptotic and proliferative signaling cascade within the body, with the mammalian target of rapamycin complexes 1 and 2 (mTORC1/2) and glycogen synthase kinase 3 (GSK3) playing significant roles in the healing process of DFUs (55, 56). Notoginsenoside Ft1 has been shown to enhance VEGF expression via the PI3K/Akt/mTOR signaling pathway, thereby promoting the formation of vascular and granulation tissues, controlling blood sugar, and expediting epithelialization (57). Additional pathways implicated in angiogenesis include the transforming growth factor-beta (TGF-β)/Smad signaling pathway, AMP-activated protein kinase (AMPK), and the HIF-1α/VEGF pathway, all of which facilitate angiogenesis by upregulating VEGF expression, ultimately contributing to wound healing (58–61). Nonetheless, the composition of TCM is intricate, and existing studies predominantly focus on individual components of TCM, often lacking comprehensive clinical data for validation. Future research should consider employing multi-omics approaches to explore these effects further.

### Reduce oxidative stress

Wound healing is intricately linked to oxidative stress. The nuclear factor erythroid 2-related factor 2/antioxidant response element (Nrf2/ARE) pathway facilitates the transport of antioxidants, including glutathione (GSH) and heme oxygenase-1 (HO-1), to mitigate reactive oxygen species (ROS) and suppress oxidative stress (62). TCM components, such as (-)-epigallocatechin-3-gallate (EGCG), flavonoids like Rutin and Luteolin, and Huangbai liniment (HB), have been shown to activate Nrf2, thereby reducing cellular damage and apoptosis induced by oxidative stress, and consequently enhancing wound healing in diabetic rat models (63–66). However, these investigations have only superficially addressed the underlying mechanisms and lack comprehensive validation through animal models or clinical trials.

### Regulate inflammatory responses

The NF-κB signaling pathway plays a crucial role in mediating inflammatory responses by inducing the expression of inflammatory cytokines such as TNF-α, IL-1, and IL-6, which contribute to sustained inflammation and delayed healing of diabetic wounds (67). Both Kirenol and the natural phenolic compound salicylic acid (SA) have been demonstrated to inhibit the NF-κB pathway, thereby reducing the levels of interleukin-1β (IL-1β), interleukin-2 (IL-2), IL-8, and TNF-α, ultimately modulating inflammation (68, 69). Research by Sun et al. has further indicated that paeoniflorin (PF) can downregulate inflammatory mediators and facilitate wound healing in diabetic rat models (70). The recruitment of macrophages is linked to the Notch signaling pathway, which affects macrophage-mediated early inflammatory responses. EGCG has been shown to inhibit Notch signaling, promoting the conversion of macrophages to the M2 phenotype, thereby enhancing wound healing in diabetic mice (71, 72). Moreover, the Qizhi Jiangtang Capsule (QJC) has been reported to modulate inflammatory responses by inhibiting the mitogen-activated protein kinase (MAPK) pathway (73). Despite these promising findings, current research on these TCMs is predominantly limited to animal studies, necessitating further clinical trials to substantiate their efficacy in humans.

## Classification of traditional Chinese medicine

TCM can be divided into systemic and topical applications based on the method of administration. Different types of TCM promote the healing of diabetic foot ulcers through different mechanisms (**Table 1**).

**Table 1:** Different traditional Chinese medicine for treating diabetic foot ulcers.

Study	Chinese medicine	Administration method	Model	Mechanism
Ko C H, et al., 2014 (74)	Astragali Radix, Radix Rehmanniae	Oral	Human	Restore fibrosis formation, promote angiogenesis, and control inflammation.
Soleimani Z, et al., 2017 (75)	flaxseed oil omega-3 fatty acids supplementation	Oral	Human	Improves insulin sensitivity, reduces oxidative stress, and inflammatory responses
Zhao B, et al., 2021 (79)	Quercetin	Gavage	Diabetic rats	Inhibits inflammatory responses and protects nerves
Liu M, et al., 2019 (80)	Berberine	Injection	Diabetic rats	Inhibits the expression of proinflammatory cytokines (TNF-α, IL-6, IL-1β)
Zhang B, et al., 2018 (81)	Tanshinone IIA	Injection	Diabetic rats	Inhibits the expression of pro-inflammatory cytokines (TNF-α, IL-6, IL-1β) and increases the expression of anti-inflammatory factor IL-10.
Liu Y, et al., 2020 (83)	Cortex Phellodendri Compound Fluid	Topical	Human	Provides serum growth factor (VEGF, EGF, BFGF) levels to promote angiogenesis
Li S, et al., 2011 (84)	Tangzu Yuyang Ointment	Topical	Human	Diabetic foot ulcers heal faster, but blood sugar doesn’t improve
Abdoli A, et al., 2022 (85)	olive oil	Topical	Human	Accelerated healing of diabetic foot ulcers.
Xiaolan X, et al., 2019 (86)	Shenghong wet dressing	Topical	Human	Control inflammation and inhibit bacteria
Fallah Huseini H, et al., 2024 (91)	Teucrium polium	Topical	Human	Accelerated healing of diabetic foot ulcers.
Huang Y, et al., 2021 (92)	Plectranthus amboinicus, Centella asiatica	Topical	Human	Promote the transformation of M1 macrophages into M2 macrophages to promote wound healing.
Mohajeri G, et al., 2014 (93)	Kiwifruit	Topical	Human	Promotes angiogenesis and epithelialization of wounds
Sun X, et al., 2014 (97)	Shengji ointment, bromelain	Topical	Human	Expand blood vessels, improve blood flow to the wound, and promote granulation tissue growth.

### Systematic traditional Chinese medicine

TCM, as the traditional medicine of China, has a history of several thousand years. Oral Chinese herbs have shown better efficacy for numerous diseases, including the treatment of diabetic foot ulcers. Chun et al. found that a Chinese herbal formula (NF3) consisting of Astragalus and Radix et Rhizoma Dioscoreae could reduce inflammation and promote wound healing (74). Similarly, oral intake of Centella asiatica extract or omega-3 has been shown to expedite the healing process of diabetic foot ulcers (75, 76). Conversely, a study conducted by Mehrdad Mokhtari et al. revealed that while curcumin effectively lowered fasting blood glucose levels and enhanced antioxidant capacity in patients, it did not significantly accelerate wound healing (77). Numerous studies have substantiated the efficacy of herbs in inhibiting apoptosis, combating oxidative stress, reducing inflammatory factors, and protecting neural nutrients (78–82). Despite these promising findings, it is important to note that the majority of these studies remain at the preclinical stage, primarily involving animal models, and the overall quality of the literature is not consistently high. Moreover, the diabetic rat model cannot fully simulate the complex pathology of DFUs, and more clinical translational validation is needed in the future.

### Traditional Chinese medicine for external application

The topical application of herbal formulations appears to be a more direct and effective method compared to herbal decoctions. The compound cypress oil solution, which includes forsythia, cypress, honeysuckle, dandelion, and centipede, has been shown to reduce inflammatory responses and enhance growth factor activity (66). In a controlled trial, this solution was found to increase growth factor concentrations and promote the healing of ulcer wounds more effectively than standard care (83). In a prospective randomized controlled trial conducted by Shufa Li et al., the healing rate of compound TANGZU YUYANG Ointment (TYO) for diabetic foot ulcers was reported to be 79.2%, demonstrating significant treatment efficacy with minimal side effects (84). Furthermore, herbal dressings derived from these formulations have also yielded promising outcomes (85, 86). The topical application of Chinese herbal medicine not only mitigates inflammation but also stimulates fibroblast proliferation, collagen synthesis, keratinocyte migration, and neovascularization, ultimately facilitating wound healing (87, 88). Topical application of TCM can also play an antibacterial role in bacterial infections that are often associated with diabetic foot ulcers, including Pseudomonas aeruginosa, Staphylococcus aureus (MRSA), Pneumococcus, Streptococcus, and so on (84, 89, 90). There are more trials of topical application of herbs than oral administration of herbs that have confirmed their efficacy (91–97). The beneficial effects of TCM in the local treatment of DFUs are attributed to stimulating cell proliferation, inhibiting local inflammatory responses, and promoting angiogenesis through increased vascular growth factors (VEGF, PDGF, etc.) (98).

## Discussion

Diabetic foot ulcers present a complex pathogenesis and necessitate comprehensive treatment strategies. TCM, characterized by its multi-target and multi-level actions, offers various therapeutic approaches for DFUs. At present, the majority of research concerning the mechanisms underlying TCM treatment for DFUs remains at the preclinical stage. Despite notable advancements, diabetic foot rat models inadequately replicate the pathological complexity of DFUs, highlighting the need for future validation through experiments utilizing human-like skin models. Furthermore, although current research has demonstrated that TCM can facilitate wound healing via multiple pathways, most studies have concentrated on single-mechanism pathways. The application of spatial multi-omics technologies could enhance the exploration of TCM’s characteristics in treating DFUs through multi-targeted mechanisms. Moreover, existing studies on TCM treatment for DFUs are often limited by small sample sizes and suboptimal research designs, leading to evidence of generally low quality. And, there is a paucity of studies examining the effects of TCM dosage and administration methods on treatment efficacy. To address these limitations and enhance clinical application, future research should encompass multicenter, large-scale randomized controlled trials.

## Conclusion

In summary, while TCM has demonstrated some efficacy in the management of DFUs, substantial progress is required to elucidate its efficacy and mechanisms of action. Additionally, exploring their integration with Western medicine may offer a more comprehensive solution to the global challenge posed by DFUs.
